# Supplementary material for: Degradation of Carbendazim by Molecular Hydrogen on Leaf Models
Source: Plants (Basel). 2022 Feb 25;11(5):621. doi: 10.3390/plants11050621 (PMC8912477; doi:10.3390/plants11050621)
Supplement: Supplementary file 1 [file plants-11-00621-s001.zip › plants-1608441-supplementary.pdf]

Supporting Information

This file includes:

Supplementary materials and methods

Table: S1

Figures: S1-S6

References: 8

Pages: 14

Supplementary materials and methods

Broad-spectrum experiment of CAR degradation

Radish (*Raphanus sativus* L. cv. Yanghua), cucumber (*Cucumis sativus* L. cv. Lufeng), rice (*Oryza sativa* L. cv. Wuyujing No.3), rapeseed (*Brassica napus* L. cv. Zhongshuang No.11), alfalfa (*Medicago sativa* L. cv. Victoria), and pepper (*Capsicum annuum* L. Hongwo No.8) were used in broad-spectrum experiment. The identical five-days-old seedlings were sown in flowerpot containing a mixture of peat and vermiculite (3:1; v/v) and cultivated in a growth chamber (Ningbosaifu PRX-450C, Ningbo Saifu Experimental Instrument Co., Ltd., Zhejiang, China) with a light intensity of 200 μmol m^{-2} s^{-1} at 14/10 h (light/dark) photoperiod for 6 weeks.

To test whether the molecular hydrogen-regulated CAR degradation is relatively universal (Figure S2), CAR solution at a concentration of 10.46 mM was applied in radish, cucumber, rice, rapeseed, alfalfa, and pepper, and each potted plant was sprayed with 20 mL CAR solution. After 24 h treatment, above potted plants were divided into two groups and placed in two identical trays (20-40 pots for each). The two trays were filled with 500 mL dH_2O or HRW for another 24 h.

Determination of non-protein thiol (NPT) Content

Non-protein thiol (NPT) content was determined as previously described [1]. Approximately 1 g sample was ground with 4 mL of 0.02 mM EDTA, and then centrifuged at 10000·rpm for 30 min at 4°C (Eppendorf 5804R, Hamburg, Germany). Afterwards, 0.2 mL supernatants were mixed with 2.6 mL of 150 mM phosphate buffer saline (PBS, pH 8.2) and 0.1 mL of 10 mM 5, 5'-Dithiobis-(2-nitrobenzoic acid) (DTNB), and then the mixture volume was adjusted to 10 mL by the addition of 7.1 mL of absolute methanol. After 15 min, the NPT content was determined by measuring the absorbance of the clear supernatant at 412 nm and expressed as nmol g^{-1} fresh weight (FW).

Assessment of CAR-induced phytotoxicity

Tomato seedlings at the six leaves stage were sprayed with 10.46 mM CAR. After 24 h treatment, 20 mL of different concentrations (0, 1, 5, 10, 15, and 20 mM) of GSH solution was applied to the CAR-treated plants. The sample without any treatment was the control (Con). Seedlings were used to determine photosynthetic parameters by a photosynthesis system (LI-6800, LI-COR, USA) [2]. The maximum PSII quantum yield (Fv/Fm) was measured on treated leaves after 30 min in the dark. The net photosynthetic rate (Pn) of leaves was determined by the photosynthesis system. The coefficient for photochemical quenching (qP=(Fm'-Fs)/(Fm'-Fo') was calculated [3]. Lipid peroxidation was determined in terms of thiobarbituric acid reactive substances (TBARS) content [4].

Determination of antioxidant system

H_2O_2 content of the tomato leaves was analysed according to the previous method [5]. Meanwhile, O_2^- was quantified using the method of hydroxylamine oxidation according to described method [6].

Superoxide dismutase (SOD), catalase (CAT), ascorbate peroxidase (APX), and guaiacol

peroxidase (POD) activities were determined in the tomato leaves according to the previous methods [5,7]. Approximately 0.5 g of tomato leaves were ground with 3 mL of cold 50 mM phosphate buffer solution buffer (pH 7.8) containing 0.2 mM EDTA, 2 mM ascorbate (AsA), and 2% PVP, and transferred into a centrifuge tube. The grinding fluid was centrifuged at 12000 rpm for 20 min at 4℃. After centrifugation, the supernatant was used for the determination of antioxidant enzyme activities. According to the previous method [8], the protein concentration of extracts was determined. The volume of enzymes required to result in 50% inhibition of nitro blue tetrazolium chloride monohydrate (NBT) reduction rate was defined as one unit (U) of SOD activity. The enzyme activity of CAT was determined on the basis of the rate of H_2O_2 at 240 nm decreasing for 3 min. The enzyme activity of APX was measured by monitoring the reduction in absorbance at 290 nm for 3 min. The activity of POD enzyme was determined by detecting the oxidation of guaiacol at 470 nm for 3 min.

Supplementary Table S1 The sequences primer sequences for qPCR

Primer names	Forward	Reverse
GAPDH	AGGCTGGAATTGCTTTGAG	CCAGGCCCACAAAACTAA
GR	TCTGATGCTGCCCTTGA	GCGACTCCTCGGTATGG
ABC2	AGGTTGACGATTGCAGTTGA	TCTCATCACAATTGCAGCAG
ABC3	GCACTTGTGCAGGAGTTTGT	TGTCAGCCGTTTACGTTGTT
ABC4	TCATTGAGGAGGTCATGGAA	AATCCAGAGGTTGGCTCATC
GST2	TGAATCGGTGGTAGAGGGAT	TCCATGCATGTAAACAAGGG
GST3	ATGGACAACAAAGGGAGG	CCAATCAACGCAATATCC
GST4	TGCTCTAGAATTGTTGAGGA	CGCGGATCCTGCGGTATGCC
GSH1	CTGCATTCTGGGTGGGT	CTCGGCTACTTCGTTCA
GS	AGTGGAAAGCTAGGCTGCTG	TCATCCAAGCTCCACAACCC
CYP724B2	CATACGAAGCACGAAGGA	ACCCGCTGGAATCACATA
CYP72A	TCGCATAAGGGTGAGT	CCTG AGTGGCAAGACA
GPX	CCGGAACAAATGAGGAGA	TCCACAAGGAATTTGGTG
APX1	TCTGAATTGGGATTTGCTGA	CGTCTAACGTAGCTGCCAAA
G-POD	TGATCGCGAGAAGATACCTG	ATCACCATTGGCTTCTGACA
CAT1	TGATCGCGAGAAGATACCTG	CTTCCACGTTCATGGACAAC
Cu/Zn-SOD	GTATCACAGGGCGTATGTCG	GGGCTTCATAGATTCCCAGA
DHAR	CCCTGATGTCCTTGGAGACT	AAGAACCATTTGGGCTTGTC
MDHAR	TCCGAACAAACATACCTGGA	CGTGTGTGCAGTTAGCAATG

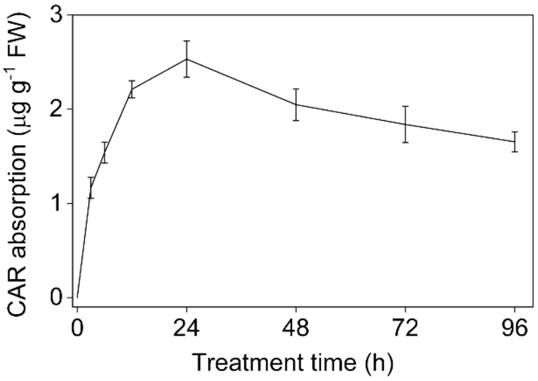

Figure S1. Time curve of CAR absorption in tomato leaves. After seedlings at the six leaves stage were treated with 10.46 mM CAR, time course of CAR absorption was measured. Error bars represent the standard deviation (SD; n=3).

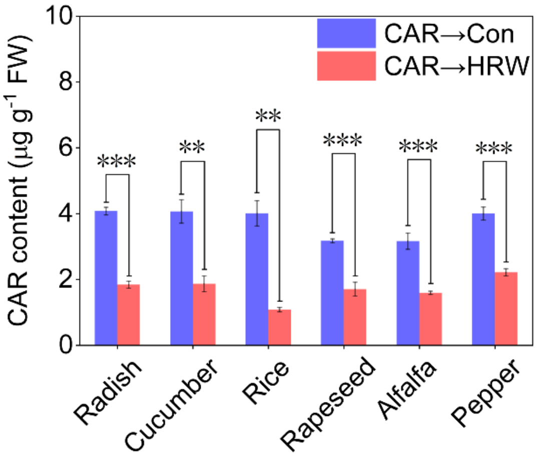

Figure S2. Molecular hydrogen-decreased CAR residues might be a universal event. Seedlings of radish, cucumber, rice, rapeseed, alfalfa, and pepper were sprayed with 10.46 mM CAR for 24 h, followed by the treatment with dH_2O (Con) or HRW for another 24 h. Afterwards, CAR content was determined. Error bars represent the standard deviation (SD; n=3). ** and *** indicate significant difference at P<0.01 or P<0.001 analyzed by independent-sample t-test.

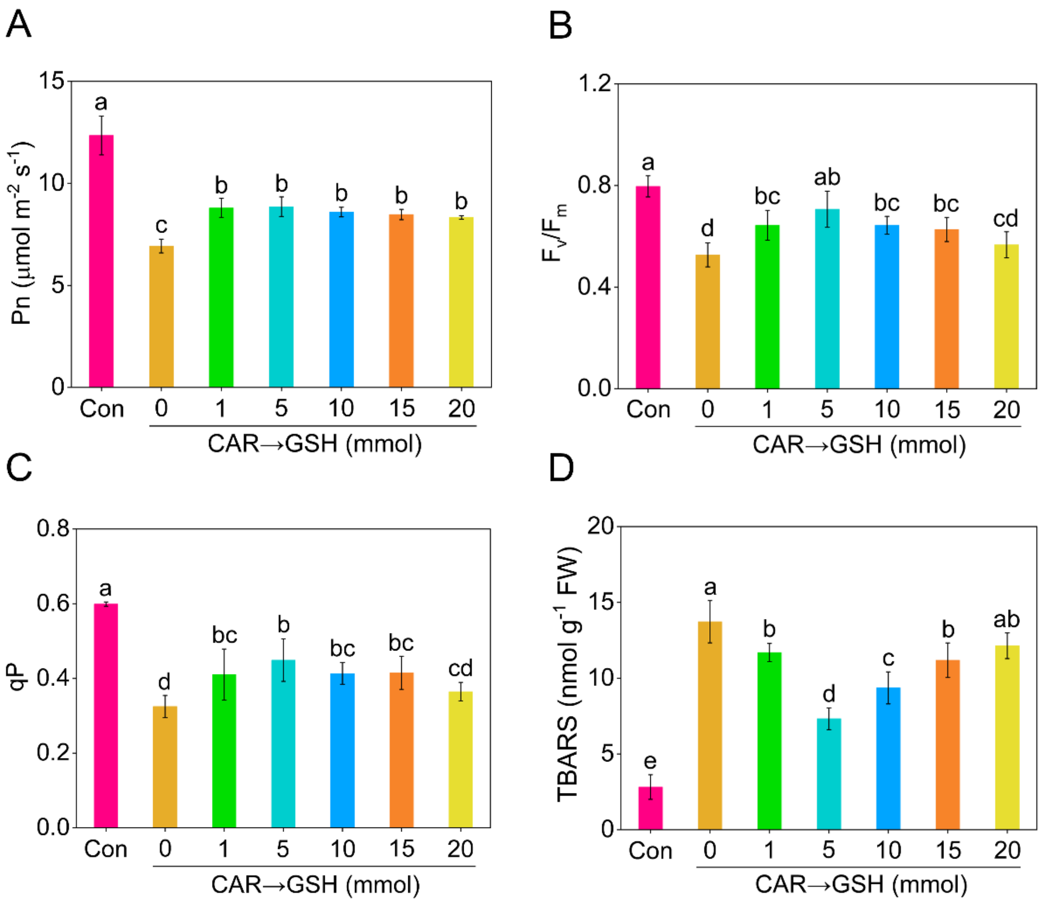

Figure S3. Changes in net photosynthetic rate (Pn; A), the maximum PSII quantum yield (Fv/Fm; B), photochemical quenching coefficient (qP; C), and thiobarbituric acid reactive substances (TBARS; D) contents in tomato seedling leaves. Seedlings at the six leaves stage were exposed to CAR for 24 h, followed by the treatment with the indicated concentrations of GSH for another 24 h. The sample without any treatment was the control (Con). Error bars represent the standard deviation (SD; n=3). Bars with different letters are significantly different (P<0.05) according to Duncan's multiple test.

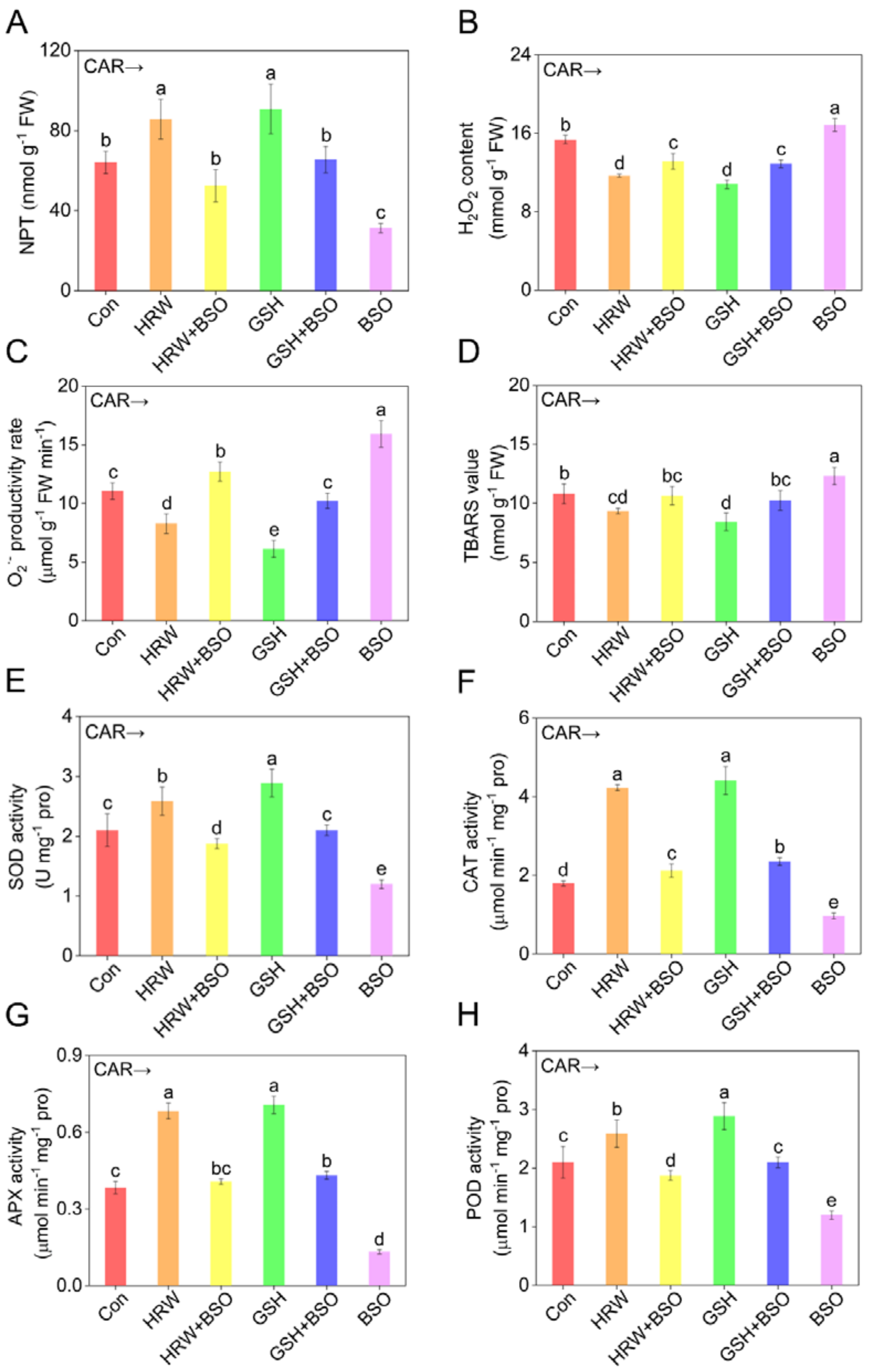

Figure S4. Changes in redox homeostasis. Tomato seedlings at the six leaves stage were exposed to CAR for 24 h, followed by the treatment with dH_2O (Con), HRW, GSH, or BSO, alone or their combinations for another 24 h. Afterwards, contents of NPT (A), H_2O_2 (B), O_2^- (C), and TBARS (D), and activities of SOD (E), CAT (F), APX (G), and POD (H) were determined. Error bars represent the standard deviation (SD; n=3). Bars with different letters are significantly different (P<0.05) according to Duncan's multiple test.

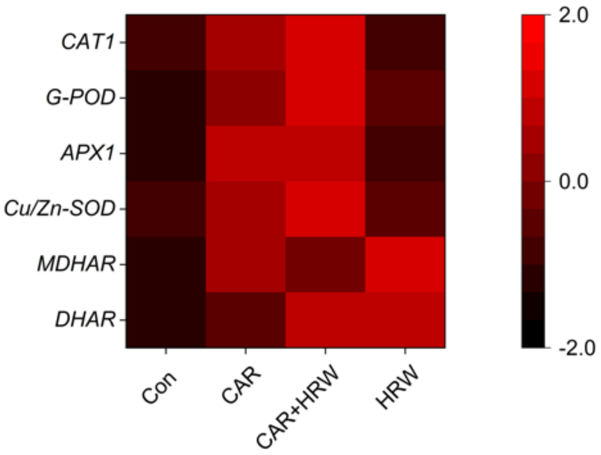

Figure S5. Comparison of expression of antioxidant genes. Tomato seedlings at the six leaves stage were respectively treated with dH_2O (Con), 10.46 mM CAR, and HRW for 48 h. CAR was applied 24 h prior to HRW application for 24 h (CAR+HRW). Afterwards, *CAT1*, *G-POD*, *APX1*, *Cu/Zn-SOD*, *MDHAR*, and *DHAR* transcript levels were analyzed by RT-qPCR. The relative expression levels of genes were normalized by log_2 transformation (n=3). Red is the relatively high expression; black is the relatively low expression.

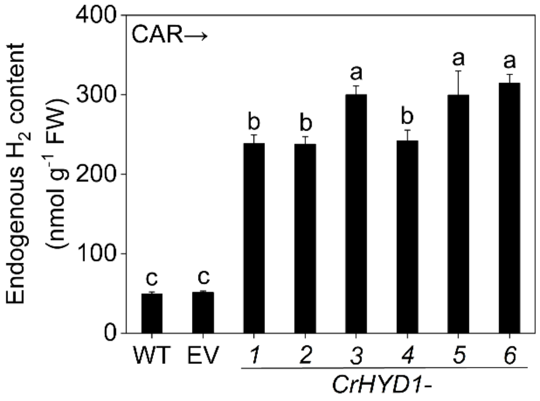

Figure S6. H_2 production in WT, EV, and six *CrHYD1* lines. After Arabidopsis seedlings were sprayed with 10.46 mM CAR for 24 h, endogenous H_2 content was measured. Error bars represent the standard deviation (SD; n=3). Bars with different letters are significantly different (P<0.05) according to Duncan's multiple test.

Reference

1. Qian, M.; Li, X.; Shen, Z. Adaptive copper tolerance in *Elsholtzia haichowensis* involves production of Cu-induced thiol peptides. *Plant Growth Regul.* **2005**, *47*, 65–73. https://doi.org/10.1007/s10725-005-1535-0
2. Gong, B.; Li, X.; Bloszies, S.; Wen, D.; Sun, S.; Wei, M.; Li, Y.; Yang, F.; Shi, Q.; Wang, X. Sodic alkaline stress mitigation by interaction of nitric oxide and polyamines involves antioxidants and physiological strategies in *Solanum lycopersicum*. *Free Radical Bio. Med.* **2014**, *71*, 36–48. https://doi.org/10.1016/j.freeradbiomed.2014.02.018
3. Schreiber, U.; Schliwa, U.; Bilger, W. Continuous recording of photochemical and non-photochemical chlorophyll fluorescence quenching with a new type of modulation fluorometer. *Photosynth. Res.* **1986**, *10*, 51–62. https://doi.org/10.1007/BF00024185
4. Su, J.; Zhang, Y.; Nie, Y.; Cheng, D.; Wang, R.; Hu, H.; Chen, J.; Zhang, J.; Du, Y.; Shen, W. Hydrogen-induced osmotic tolerance isassociated with nitric oxide-mediated proline accumulation and reestablishment of redox balance in alfalfa seedlings. *Environ. Exp. Bot.* **2018**, *147*, 249–260. https://doi.org/10.1016/j.envexpbot.2017.12.022
5. Su, J.; Nie, Y.; Zhao, G.; Cheng, D.; Wang, R.; Chen, J.; Zhang, S.; Shen, W. Endogenous hydrogen gas delays petal senescence and extends the vase life of lisianthus cut flowers. *Postharvest Biol. Tec.* **2019**, *147*, 148–155. https://doi.org/10.1016/j.postharvbio.2018.09.018
6. Gong, B.; Wen, D.; VandenLangenberg, K.; Wei, M.; Yang, F.; Shi, Q.; Wang, X. Comparative effects of NaCl and $NaHCO_3$ stress on photosynthetic parameters, nutrient metabolism, and the antioxidant system in tomato leaves. *Sci Hortic-Amsterdam* **2013**, *157*, 1–12. https://doi.org/10.1016/j.scienta.2013.03.032
7. Jin, Q.; Zhu, K.; Cui, W.; Xie, Y.; Han, B.; Shen, W. Hydrogen gas acts as a novel bioactive molecule in enhancing plant tolerance to paraquat-induced oxidative stress via the modulation of heme oxygenase-1 signalling system. *Plant Cell Environ.* **2013**, *36*, 956–969. https://doi.org/10.1111/pce.12029
8. Bradford, M.M. A rapid and sensitive method for the quantitation of microgram quantities of protein utilizing the principle of protein-dye binding. *Anal. Biochem.* **1976**, *72*, 248–254. https://doi.org/10.1016/0003-2697(76)90527-3
